# Investigation of anti-depression effects and potential mechanisms of the ethyl acetate extract of *Cynomorium songaricum* Rupr. through the integration of *in vivo* experiments, LC-MS/MS chemical analysis, and a systems biology approach

**DOI:** 10.3389/fphar.2023.1239197

**Published:** 2023-10-25

**Authors:** Xinyu Zhang, Lingling Li, Jianxin Chen, Mengyuan Hu, Yali Zhang, Xuya Zhang, Yi Lu

**Affiliations:** School of Traditional Chinese Medicine, Beijing University of Chinese Medicine, Beijing, China

**Keywords:** *Cynomorium songaricum* Rupr., ethyl acetate extract, depression, neuroprotection, microglia polarization, LC-MS/MS, network analysis

## Abstract

**Background:** *Cynomorium songaricum* Rupr. has long been used as an anti-inflammatory, antidepressant, and anti-aging agent in traditional Chinese medicine in Asia. Its ethyl acetate extract (ECS) has been identified as the main antioxidant component with neuroprotective and estrogen-like effects. However, the potential of ECS in treating depression has not been explored yet.

**Methods:** We identified the primary metabolites in ECS in this study using liquid chromatography-electrospray tandem mass spectrometry (LC-MS/MS). Network analysis was used to find the potential targets and pathways associated with the anti-neuroinflammatory depression action of the ECS. In addition, we established a corticosterone (CORT)-induced depression mouse model to assess ECS’s antidepressant effects by monitoring various behavioral changes (e.g., sucrose preference, forced swimming, tail suspension, and open field tests) and biochemical indices of the hippocampus, and validating the network analysis results. Significant pathways underwent verification through western blotting based on network analysis prediction.

**Results:** Our study demonstrates that ECS possesses significant antidepressant activity. The LC-MS/MS analysis of ECS identified 30 main metabolites, including phloridzin, phlorizin, ursolic acid, and naringenin, as well as other flavonoids, terpenoids, and phenolic acids. These metabolites were found to be associated with 64 candidate target proteins related to neuroinflammatory depression from the database, and ten hub proteins were identified through filtration: CXCL8, ICAM1, NOS2, SELP, TNF, IL6, APP, ACHE, MAOA and ADA. Functional enrichment analyses of the candidate targets revealed their primary roles in regulating cytokine production, inflammatory response, cytokine activity, and tumor necrosis factor receptor binding. *In vivo*, ECS improved hippocampal neuroinflammation in the mouse model. Specifically, ECS reduced the expression of inflammatory factors in the hippocampus, inhibited M1 microglial cell polarization, and alleviated depression through the regulation of the NF-κB-NLRP3 inflammation pathway.

**Conclusion:** Based on experimental and network analysis, this study revealed for the first time that ECS exerted antidepression effect via anti-neuroinflammation. Our research provides valuable information on the use of ECS as an alternative therapeutic approach for depression.

## 1 Introduction

Depression is a common and multifaceted mental condition resulting in sleep disorders, anhedonia (i.e., loss of pleasure or interest in activities), decreased volitional activity, or impaired cognitive function. Its profound effects on social interaction and behavioral functions pose a serious public health concern, intensifying the risk of chronic diseases like dementia, Alzheimer’s disease, and hypertension (Gracia-Gracia et al., 2023). By 2030, the World Health Organization predicts that depression will surpass cardiovascular disease as the second most common condition worldwide. Furthermore, in China, the cost of managing depression is increasing (Collins et al., 2011; Whiteford et al., 2013). Current evidence suggests that, while the origin and pathogenesis of depression remain elusive, they may involve abnormalities in monoamine neurotransmitter synthesis and release, an unbalanced hypothalamic-pituitary-adrenal (HPA) axis, abnormal neural transmission, and neural inflammation. Notably, neural inflammation has been associated with the pathophysiology of various neurological disorders, necessitating further investigation (Aleksandrova et al., 2020). Although many contemporary antidepressants exert pharmacological effects by inhibiting neuroinflammation, they typically cause withdrawal syndrome, upper gastrointestinal hemorrhage, and mucositis (Ling et al., 2022). Thus, there is a demand for antidepressants that are more novel, safer, and more effective. Traditional Chinese medicines that contain various low-molecular-weight substances are gaining popularity due to their ability to bind and modulate various proteins to achieve desired therapeutic outcomes with minimal adverse effects (Zhang et al., 2022).

*Cynomorium songaricum* Rupr. is an herb that reportedly has renoprotective properties, improves immune function, and relieves fatigue. In the early stages of our research, the antioxidant effects of various *Cynomorium songaricum* Rupr. extracts were assessed. We discovered that the ECS has antioxidant and estrogen-like effects as well as improve cognitive synapse-related protein expressions in chronically stressed mice after castration. It shows a protective effect on hippocampal neurons, which results in improving cognitive dysfunction caused by chronic stress (Lu et al., 2012a; Ma et al., 2017; Tian et al., 2019). Therefore, in this study, it is proposed that ECS exerts antidepressant effects by attenuating neuroinflammation via neuroprotective mechanisms.

**FIGURE 1:**
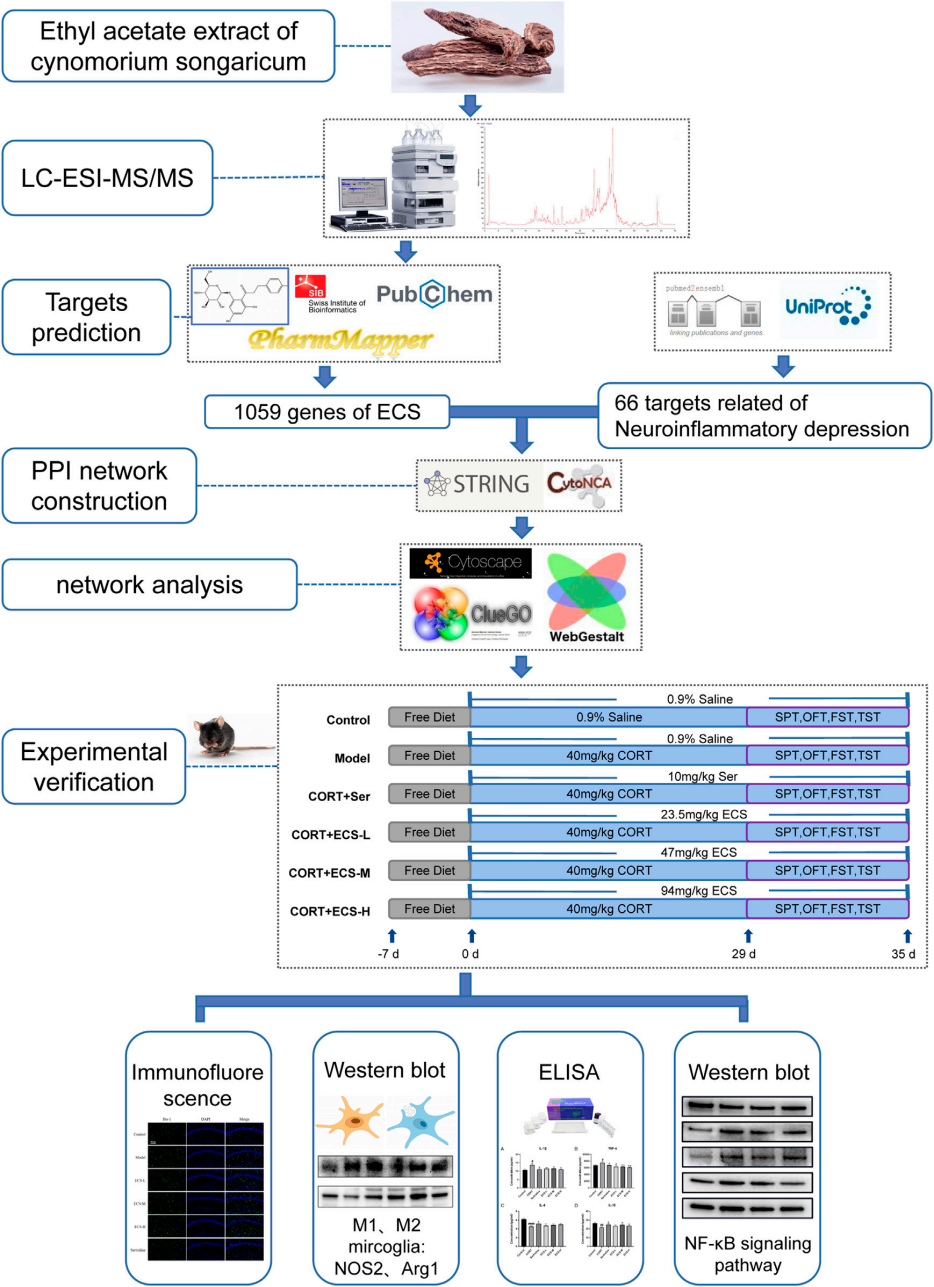
Technical Strategy of the current study.

In this study, we used network analysis and LC-MS/MS to predict the primary metabolites and their targets in ECS and to investigate its antidepressant mechanism of action. First, the primary metabolites of ECS were identified and screened using LC-MS/MS combined with the Traditional Chinese Medicine Systems Pharmacology Platform (TCMSP) and a literature review. The SwissTargetPrediction network server was then used to predict the active metabolites targets, construct active metabolite-target and protein interactions, and analyze ECS’s pharmacodynamic basis and mechanism. Finally, ECS’s pharmacodynamic effects and key targets in a CORT-induced depression mouse model were validated. Figure 1 illustrates the research design.

## 2 Materials and methods

### 2.1 Drugs and reagents

Samples of the Chinese traditional medicinal plant *Cynomorium songaricum* Rupr. were obtained from the pharmacy of Guo yi tang, Beijing University of Chinese Medicine (Catalog number 701002539) and stored in a ventilated and dry location. Sertraline was purchased from Guang’anmen Hospital, China Academy of Chinese Medical Sciences (Beijing, China) (Zhejiang Jingxin Pharmaceutical Co., Ltd., National Medicine Standard H20051076; specification: 50 mg). ELISA kits of TNF-α (ELM-TNFα), IL-1β (ELM-IL1β), IL-4 (ELM-IL4), and IL-10 (ELM-IL10) were obtained from RayBiotech Life, Inc (Norcross, GA, USA). Antibodies against GAPDH (G0100) were obtained from LABLEAD BIOTECHNOLOGY CO., LTD. (Beijing, China); antibodies against Arg1 (66129-1-lg) were obtained from Proteintech Group, Inc. (Wuhan, China); antibodies against caspase 1 (sc-56036) were obtained from Santa Cruz Biotechnology, Inc (Dallas, TX, USA). Antibodies against alpha tubulin (ab176560), NOS2 (ab178945), and Iba-1 (ab178846) and FITC-labeled secondary antibodies (ab6717) were purchased from Abcam (Cambridge, UK). Antibodies against NF-κB p65 (8,242), p-NF-κB p65 (3,033), and NLRP3 (15101) were purchased from Cell Signaling Technology, Inc. (Danvers, MA, USA).

### 2.2 Qualitative dissection concerning ECS

#### 2.2.1 ECS preparation

Following a protocol developed by our laboratory (Chen et al., 2023), the powdered *Cynomorium songaricum* Rupr. pieces were sieved through a mesh sifter with a size of 60. The resulting powder was then soaked in 70% ethanol at a proportion of 1:10 (w/v) overnight. The following day, the filtrate was collected and subjected to three rounds of ultrasonic extraction. For the first extraction, 2 L of 70% ethanol was added to the filtrate, which was then subjected to ultrasonic extraction for 1 h. Afterward, the supernatant was collected, and 1 L of 70% ethanol was added for a second super-lift extraction, which lasted for 45 min. Subsequently, that supernatant was collected again; additionally, 1 L of 70% ethanol was added for a third ultrasonic extraction, which lasted for 30 min. This final supernatant was collected and subjected to rotary evaporation under heat until no alcohol taste/smell remained. The ethyl acetate polar component of the concentrated solution was then extracted with petroleum ether and ethyl acetate in a 2:1 (v/v) ratio. The ethyl acetate organic layer was then subjected to further concentration by rotary evaporation and subsequently freeze-dried for future use. The extraction rate of ECS was 2.36%, with impurities less than 2% and the total ash value less than 12%, all of which meeting the pharmacopoeia standards. Finally, the ECS powder was conserved at a temperature of −20°C.

#### 2.2.2 Sample preparation

Briefly, 1.0 g of ECS dried powder was accurately weighed and added to 50 mL of 70% ethanol solution through reflux for 1 h. Subsequently, the solution was filtered using a 0.22-µm membrane.

#### 2.2.3 LC-MS/MS analysis

LC-MS/MS dissection was performed using an Ultimate 3,000 with a Waters ACQUITY UPLC HSS T3 C18 column (2.1 mm × 100 mm, 1.8 µm; Waters Corporation, Milford, MA, USA). Investigation and dissection on high-resolution MS were performed using a Q-Exactive Orbitrap mass spectrometer MS system (Thermo Scientific, Waltham, MA, USA) with a single electron spray ionization source. Specific experimental details are provided in Supplementary Approach One.

#### 2.2.4 Identification of metabolites

Metabolites were identified by matching the molecular formulas, retention times, and MS fragmentation patterns using corresponding standard materials, references, and web databases containing Chemical Name Search^1^ and Massbank^2^.

### 2.3 Network analysis studies

#### 2.3.1 Candidate metabolites screening and target prediction

The main ECS metabolites were identified using the following criteria: MW < 500, AlogP <5, Hdon <5, and Hacc <10. They were fed into the TCMSP^3^, SwissTargetPrediction^4^ (Daina et al., 2019), SEA^5^ (Keiser et al., 2007), and PharmMapper^6^(Wang et al., 2021) for target prediction. After eliminating the duplicates, we obtained the relevant targets. Furthermore, significant targets associated with neuroinflammation and depression were obtained from the Pubmed2ensembl^7^ database. The Pubmed2 Ensembl resource, which is designed as a supplement to the BioMart mechanism for literature mining, was used to compile a list of proteins linked to depression-related neuroinflammation. The Ensembl Gene ID of GRCh37 human (*Homo sapiens*) genome assembly associated with neuroinflammation and depression was located by searching for the terms “neuroinflammation” and “depression” according to the Genome Reference Consortium.

#### 2.3.2 Venn diagram and identification of common target genes

The obtained targets were normalized in UniProt^8^, and the species was set to “human” after eliminating duplicate targets. The Venn tool^9^, an online software, was used to draw a Venn diagram to obtain the common target, which is a potential target for ECS in the treatment of neuroinflammation-related depression.

#### 2.3.3 Interaction network analysis

The analysis of protein–protein interactions (PPI) was conducted using STRING^10^ database. The resulting data were saved as TSV files and imported into Cytoscape 3.8.2. To analyze the network topology, we used CytoHubba plugin within Cytoscape. The nodes were ranked based on their network centrality using the maximal clique centrality technique.

#### 2.3.4 Functional enrichment analysis of shared targets

To detect the molecular functions (MF), biological processes (BP), and cellular components (CC) associated with the ECS-induced attenuation of depression-related neuroinflammation, these key targets were uploaded to the DAVID Bioinformatics Resources 6^11^ for Gene Ontology (GO) enrichment analysis. Subsequently, Kyoto Encyclopedia of Genes and Genomes (KEGG) enrichment was performed on these key targets to screen for putative pathways related to the ECS-mediated treatment of neuroinflammation-induced depression.

### 2.4 Experimental validation

#### 2.4.1 Animals

Male SPF C57BL/6 mice aged 7 weeks were obtained from Wei tong Lihua Biotechnology Co., Ltd. (NO.SCXK (jing) 2021–0006, China). Each animal was housed in an SPF habitat. A 12-h light–dark cycle was maintained for all animals. Food and water were readily available throughout the study, except during the sucrose preference test. A constant temperature of 25°C ± 1°C was maintained, with a relative humidity of 55%–65% and continuous indoor air circulation. All animal experiments were performed in compliance with the regulations of the Beijing University of Traditional Chinese Medicine’s Committee for Animal Care and Use of Laboratory Animals (approval number BUCM-4-2021083003-3086).

#### 2.4.2 Assignment of groups and construction of the depression model

After a 7-day acclimatization period, the mice were randomly and equally divided into six groups (*n* = 10): control, model, sertraline, medium-dose ECS (ECS-M), high-dose ECS (ECS-H), and low-dose ECS (ECS-L) groups. CORT was dissolved in normal saline with 0.1% Tween-80% and 0.1% DMSO to create a 2 g/L solution which was then used to prepare the CORT injection. This suspension was evenly mixed using ultrasonic waves. During the experiment, each mouse that was not part of the control group was administered a daily injection of CORT at a dose of 40 mg/kg/day for 5 weeks. Mice in the control group were administered normal saline injections at the same dose and time.

#### 2.4.3 Drug treatment

The ECS freeze-dried powder was completely dissolved in an appropriate amount of DMSO and then diluted with ddH2O to a concentration of 4.7 mg/mL. In the ECS-L, ECS-M, and ECS-H groups, the medications were administered by gavage at doses of 23.5 mg/kg, 47 mg/kg, and 94 mg/kg, respectively. The operators dissolved ECS in ddH2O at a concentration of 1 mg/mL. Medication was administered intragastrically (i.g.) before CORT injections during the first week of treatment. The groups were treated as follows: 1) distilled water (10 mL/kg body weight) was the only treatment given to the control mice; 2) the Model group received distilled water i.g. (10 mL/kg body weight), and depression was induced via the CORT injection protocol; 3) mice in the sertraline group received sertraline i.g. (10.0 mg/kg/day) and CORT injection; 4) mice in the ECS-H group received ECS i.g. (94 mg/kg/day) and CORT injection; 5) mice in the ECS-M group received ECS i.g. (47 mg/kg/day) and CORT injection; and 6) mice in the ECS-L received ECS i.g. (23.5 mg/kg/day) and CORT injection.

#### 2.4.4 Behavioral evaluation

*Body weight*: The mice were weighed weekly using an electronic scale, and their corresponding growth ratio (%) was figured out as below:

$$\text{Relative growth ratio }\left(\%\right)=\frac{\left(\text{weight}\;\text{after}\;\text{modeling}-\text{weight}\;\text{before}\;\text{modeling}\right)}{\text{weight}\;\text{before}\;\text{modeling}}\times 100\;\%$$

*Sucrose preference test (SPT):* Two steps were required to complete the sucrose preference test. The mice were treated with two identical vials of 1% sucrose solution for 24 h during the adaptation period. One sucrose solution was then substituted with water, and after 12 h, the positions of both bottles are switched to avoid position preference, and the animals are fed for an additional 12 h. Then, the operators deprived the mice of water and food, lasting for 24 h. In next step, those animals were exposed to a sucrose solution and a normal water solution for 4 h. The volume of every bottle was recorded before and after this test. The test was primarily used to assess the level of anhedonia in the animals (Bolaños et al., 2008).

The value of the sucrose preference (SP) is able to be figured out as follows:

$$\text{SP }\left(\%\right)=\text{sucrose intake }\left(\text{vol}\right)\;/\;\left[\text{sucrose intake }\left(\text{vol}\right)+\text{water intake }\left(\text{vol}\right)\right]\times 100\%$$

*Open field test (OFT):* To start with, those mice were placed in the center of an open field machine; additionally, they were allowed to explore freely for 3 min. After mice adapting, the operators recorded the distance traveled by every mouse over next 3 min. The operators cleaned the open field device using 75% ethanol before assessing the following mouse. And the observers was not sure about the allocation of every mouse (Song et al., 2020).

*Forced swimming test (FST)*: A 18 cm × 30 cm hyaline cylinder was full of water 20 cm deep at 25°C ± 1°C, and the total mice were separately placed into the water for 6 min. The cumulative standstill time for each mouse in the last 4 min of the test was recorded. After every test, the water was changed. Those observers was not sure about the team allocation of every mouse (Choi et al., 2017).

*Tail suspension test (TST):* With sticky tape put roughly 1 cm from the tail tip, every mouse was suspended above the ground, lasting for 6 min. The total standstill period for the final 4 min of the experiment was assessed after the first 2 min of conditioning. When mice stop scurrying and just move to breathe, they are regarded as static. The groupings of mice were hidden from the observers (Gu et al., 2021).

**FIGURE 2:**
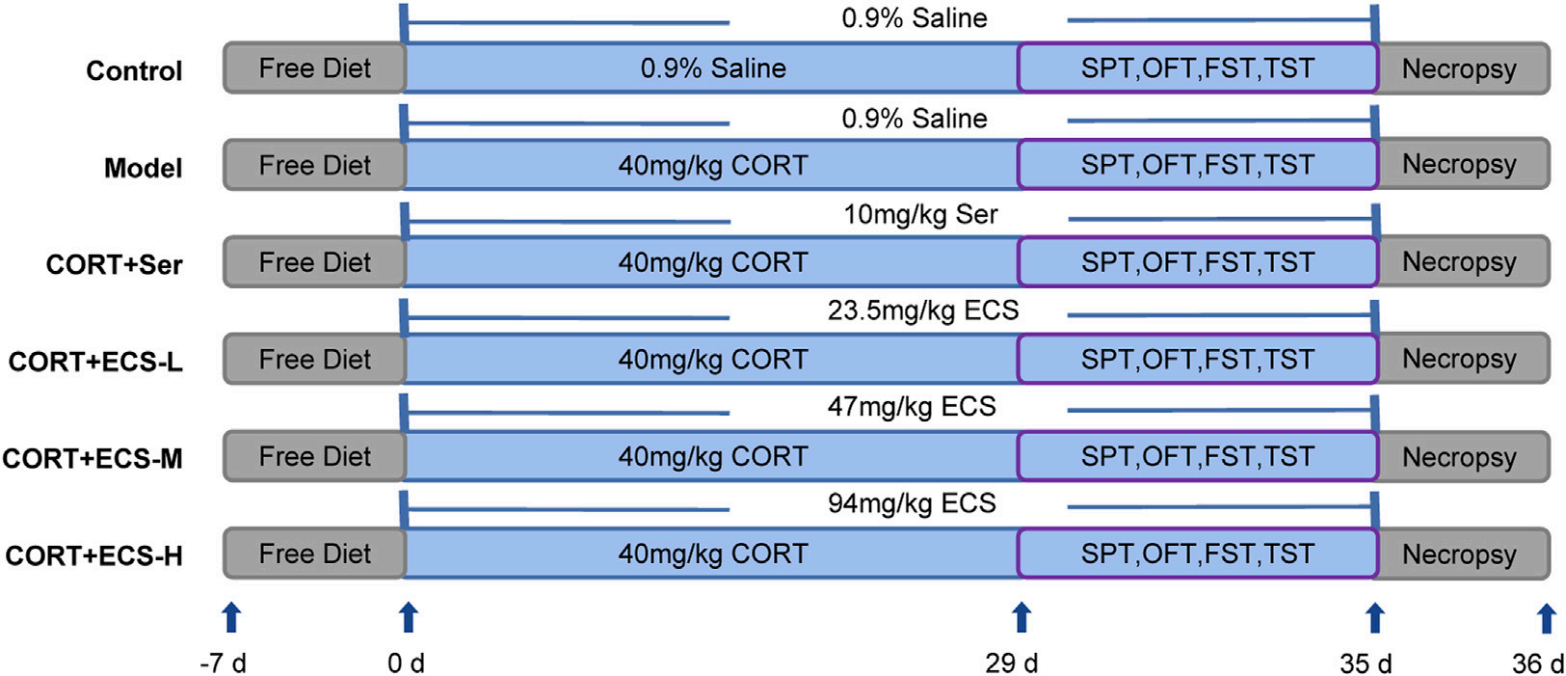
Experimental design.

The experimental procedure implemented for the CORT model and treatment is shown in Figure 2.

### 2.5 Histopathologic examination

Before all behavioral experiments ended, three mice from each group were randomly selected, anesthetized with pentobarbital sodium, and underwent cardiac perfusion. Subsequently, the brain tissue was obtained and fixed in 4% paraformaldehyde for 24 h for immunofluorescence detection of hippocampal Iba-1 protein expression. The bilateral hippocampi of the remaining mice were stored in a cryodepository tube at −80°C to analyze the expression of the relevant proteins within the subsequent hippocampal tissues.

#### 2.5.1 Immunofluorescence

After fixation in a 4% paraformaldehyde solution for 24 h, continuous coronal sections with a thickness of 5 µm were subjected to alcohol gradient dehydration and paraffin embedding. To retrieve the antigen, the sections were immersed in a boiling citric acid repair solution and then rinsed thrice using PBS (pH 7.4) for 5 min each. The sections were then incubated overnight at 4°C with a primary antibody against Iba-1 (1:500, Abcam, MA, USA). On the following day, these sections were rinsed with PBS and then treated with drops of an FITC-conjugated secondary antibody (1:3,000, Abcam, MA, USA), followed by incubation for 50 min at room temperature and away from light. After restaining with DAPI (1:100, Solarbio, Beijing, China) for 10 min and cleaning with PBS, an anti-fluorescence quenching sealing agent was applied in the dark. The entire slide was scanned using a fluorescence microscope (Leica, DMI8), and the images were using Caseviewer 2.4 software (Liu et al., 2021; Fang et al., 2022).

#### 2.5.2 ELISA

Tissues from the mouse hippocampus were homogenized using RIPA lysate (Ebifan Biotechnology Corporation, Beijing, China). The supernatant was obtained through centrifugation at 12,000 rpm for 10 min at 4°C. Commercial ELISA kits were used to measure levels of IL-4, IL-10, IL-1β, and TNF-α, following the manufacturer’s instructions. A GloMax microplate reader (Promega, Madison, WI, USA) was used for assessing the absorbance at 450 nm, and the resulting optical density values were used to calculate the expression levels of IL-4, IL-10, IL-1β, and TNF-α. To reduce inter-assay variations, all samples were assessed thrice using the same test.

#### 2.5.3 Western blot analysis

Whole proteins in the hippocampus were extracted using a RIPA lysis buffer with 1× protease inhibitor cocktail. A BCA assay test kit was used to assess the protein concentration, following the manufacturer’s instructions. In brief, the proteins were separated using a 10% SDS-PAGE gel and then transferred to a 0.45-µm PVDF membrane (Millipore, Billerica, MA, USA). These membranes were incubated at 23°C for 1 h with 5% non-fat dried milk, followed by incubation with primary antibodies against NOS2 (1: 500, Abcam, MA, USA), Arg (1: 5,000, Proteintech, Wuhan, China), alpha tubulin (1: 5,000, Abcam, MA, USA), NF-κB P65 (1:1000, Cell Signaling Technology, USA), p-NF-κB P65 (1:1000, Cell Signaling Technology, USA), NLRP3 (1:1000, Cell Signaling Technology, USA), caspase-1 (1:500, Santa Cruz, USA), and GAPDH (1:2000, LABLEAD, Beijing, China) overnight at 4°C. Following three TBST washes, the membranes were incubated at 25°C for 1 h with the appropriate goat anti-rabbit IgG-HRP secondary antibody or goat anti-mouse IgG-HRP secondary antibody (IgG-HRP) (both 1: 10000, Proteintech, Wuhan, China). Following another TBST wash, the ECL reagent was used to detect immunoreactivity. The membranes were scanned using ChemiDoc Imagers (BioRad, Hercules, CA, USA). Simultaneously, the intensity of the bands was analyzed using ImageJ software (National Institutes of Health, Bethesda, MD, USA) (Cao et al., 2022).

### 2.6 Statistical analysis

Statistical analysis was conducted using SPSS 20.0 (IBM, Chicago, IL, USA with a two-tailed Student’s t-test or one-way ANOVA followed by a *post hoc* Tukey–Kramer test. GraphPad Prism 6.0 was used to create figures (San Diego, CA, USA). Quantitative data are presented as means ± SEM, with statistical significance was set at *p* < 0.05.

## 3 Results

### 3.1 Chemical profiling of ECS

**FIGURE 3:**
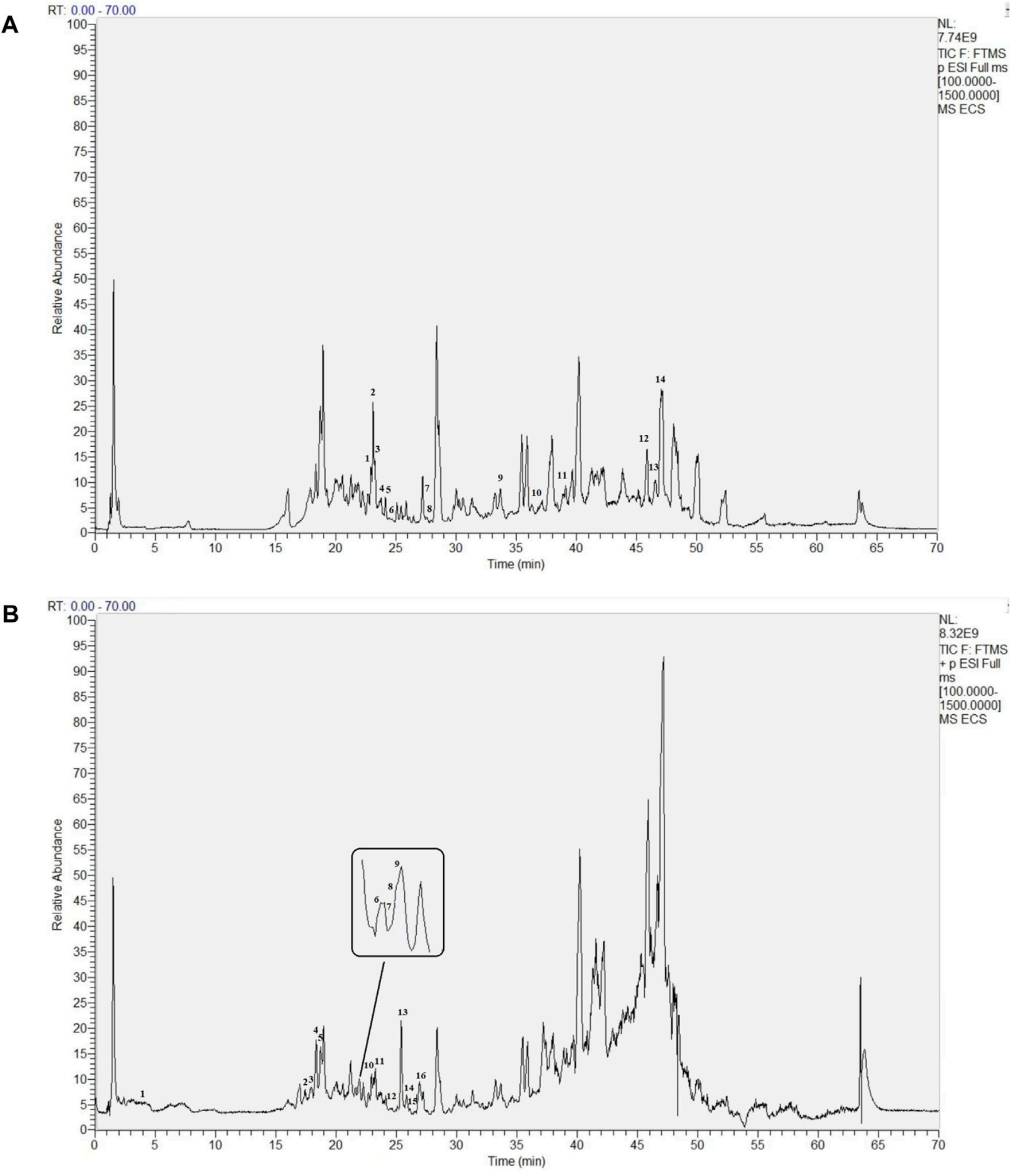
Base Peak intensity Chromatograms of Ecs acquired by Lc- Ms**(A)** Positive mode **(B)** Negative mode.

ECS metabolites were characterized via LC-MS/MS in both positive and negative ion modes. After analyzing the raw figures, we applied the predefined criteria (MW < 500, AlogP <5, Hdon <5, and Hacc <10) to identify a total of 30 main metabolites, which were further verified based on their relative molecular mass and MS fragmentation (Figure 3). The identified metabolites included 13 flavonoids, 8 phenols, 5 terpenoids, 1 organic acid, 2 lactones, and 1 xanthone (Table 1).

**TABLE 1:** Main metabolites identified in ECS.

Peaks	Retention time	Molecular	Adduct	Observed	Typical MS2 ions fragments (*m/z*)	Identification	Class	Sources
	(min)	Formula						
1	4.03	C_4_H_6_O_4_	[M-H]^-^	117.0194	-	Succinic acid	Organic acids	Safonova et al. (2015)
2	17.88	C_7_H_6_O_3_	[M-H]^-^	137.0244	109.0294	Salicylic acid	Phenols	Guo et al. (2017)
3	18.35	C_30_H_26_O_12_	[M-H]^-^	577.1354	425.0882, 407.0757, 289.0710, 137.0244, 125.0243	Procyanidin B2	Flavonoids	Chen et al. (2020)
			[2 M-H]^-^	1155.2772				
4	18.61	C_10_H_10_O_4_	[M-H]^-^	193.0506	175.0612, 115.9207	Isoferulic acid	Phenols	Guo et al. (2022)
5	18.72	C_30_H_26_O_12_	[M-H]^-^	577.1354	407.0753, 289.0718, 125.0242	Procyanidin B1	Flavonoids	Chen et al. (2023)
6	21.76	C_22_H_18_O_10_	[M-H]^-^	441.0829	289.0717, 169.0142, 125.0242,	(−)-Catechin gallate	Phenols	Bastianetto et al. (2009)
			[2 M-H]^-^	883.1727				
7	21.83	C_13_H_8_O_6_	[M-H]^-^	259.0248	-	Tetrahydroxyxanthone	Xanthones	TCMSP
8	21.86	C_21_H_20_O_12_	[M-H]^-^	463.0884	300.0261, 284.0315	Quercetin-3β-D-glucoside	Flavonoids	Su et al. (2023)
9	21.89	C_14_H_6_O_8_	[M-H]^-^	300.999	283.1303, 229.0142	Ellagic acid	Phenols	Zhu et al. (2022)
1	22.92	C_21_H_20_O_11_	[M + H]^+^	449.1079	287.0544	Kaempferol-7-O-β-D-glucopyranoside	Flavonoids	Lu et al. (2012b)
10	23.02	C_30_H_24_O_12_	[M-H]^-^	575.1197	447.0934	Procyanidin A2	Flavonoids	Chen et al. (2020)
2	23.12	C_22_H_22_O_11_	[M + H]^+^	463.1236	301.0703, 284.0580	Tectoridin	Flavonoids	Chen et al. (2023)
11	23.28	C_21_H_24_O_10_	[M-H]^-^	435.1298	273.0769, 167.0345	Phloridzin	Flavonoids	Kamdi et al. (2021)
3	23.28	C_15_H_14_O_5_	[M + H]^+^	275.0914	169.0496	Phloretin	Flavonoids	Barreca et al. (2017)
4	23.56	C_20_H_22_O_4_	[M + H]^+^	327.1591	118.0863	Dehydrodiisoeugenol	Phenols	Qiburi et al. (2022)
5	24.14	C_12_H_14_O_2_	[M + H]^+^	191.1068	173.0963, 145.1013	Ligustilide	Lactones	Mao et al. (2022)
6	24.42	C_14_H_12_O_3_	[M + H]^+^	229.0862	186.1491	Resveratrol	Phenols	Zhou et al. (2021)
12	25.18	C_15_H_12_O_6_	[M-H]^-^	287.0562	152.0981	Eriodictyol	Flavonoids	Li et al. (2022)
13	25.66	C_15_H_10_O_6_	[M-H]^-^	285.0406	132.8679	Luteolin	Flavonoids	Nabavi et al. (2015)
14	25.87	C_21_H_22_O_9_	[M-H]^-^	417.1192	255.0663	Liquiritin	Flavonoids	Sun et al. (2010)
15	26.52	C_15_H_8_O_7_	[M-H]^-^	299.0198	271.0612	Demethylwedelolactone	Phenols	Morel et al. (2023)
16	27.23	C_15_H_12_O_5_	[M-H]^-^	271.0612	151.0037	Naringenin	Flavonoids	Nouri et al. (2019)
7	27.53	C_15_H_10_O_5_	[M + H]^+^	271.0602	154.9903	Apigenin	Flavonoids	Nabavi et al. (2018)
8	27.61	C_15_H_22_O_2_	[M + H]^+^	235.1694	-	Artemisinic acid	Terpenoids	Ding et al. (2022)
9	33.72	C_15_H_24_O_2_	[M + H]^+^	237.185	-	Dihydroartemisinic acid	Terpenoids	TCMSP
10	36.35	C_16_H_26_O_2_	[M + H]^+^	251.2006	-	Clareolide	Terpenoids	TCMSP
11	39.27	C_12_H_16_O_2_	[M + H]^+^	193.1224	175.1118, 147.1169, 137.0597	Senkyunolide A	Lactones	Gong et al. (2022)
12	45.83	C_10_H_8_O_3_	[M + H]^+^	177.0546	149.0598, 121.1012	7-Methoxycoumarin	Phenols	Santibáñez et al. (2023)
13	46.30	C_15_H_24_O_2_	[M + H]^+^	237.1848	135.1166	Curdione	Terpenoids	Li et al. (2017)
14	46.86	C_30_H_48_O_3_	[M + H]^+^	457.3678	-	Ursolic acid	Terpenoids	Mirza et al. (2021)

### 3.2 Network analysis studies

#### 3.2.1 Potential targets of ECS in depression-associated neuroinflammation

**FIGURE 4:**
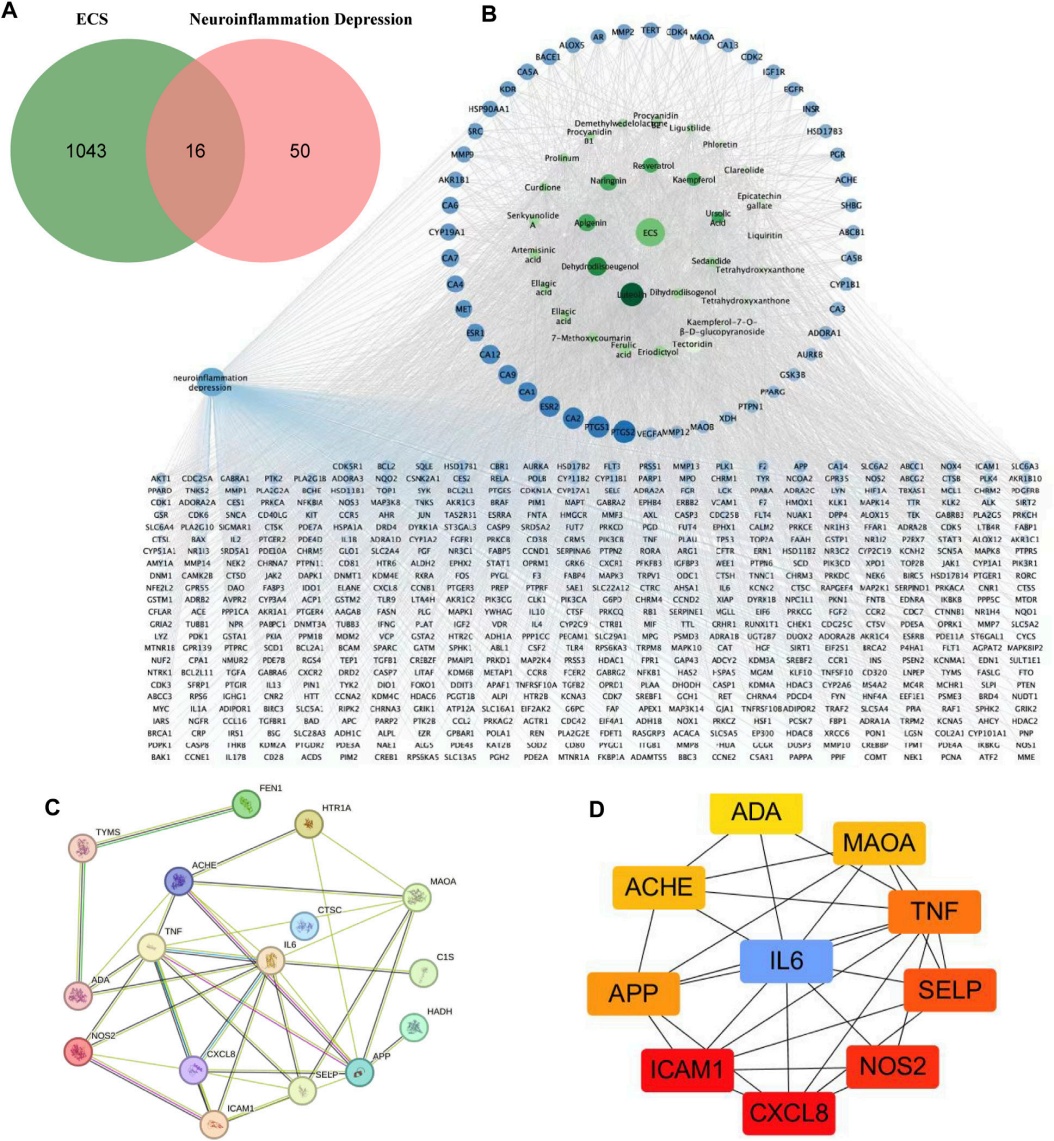
Potential targets of ECS components anti-neruoinflammation depressin were predicted by network pharmacology analysis **(A)** the venn diagram of ECS for neuroinflammation depressin treatment **(B)** the darker the color of the ECS component-disease target interaction diagram the greater the degree value and the more important the node the blue circles represent targets and the green circles represent compounds **(C)** PPI network of the 16 common targets between ECS and neuroinflammation depression **(D)** the Top 10 target network are shown.

The 2D structures of these ECS metabolites were obtained from PubChem. After eliminating duplicates, 1,059 targets were obtained. We systematically obtained 66 neuroinflammatory proteins related to depression via text mining using the Pubmed2 Ensembl database, which links more than 2 million studies to approximately 150,000 genes (Baran et al., 2011). Supplementary Table S1 provided a list of these 66 proteins along with their corresponding gene symbols and Ensembl genes. To identify the expected targets, Venn diagrams were used to map ECS-related targets to neuroinflammatory depression-linked targets (Figure 4A).

#### 3.2.2 PPI network and neuroinflammation-related targets in ECS

As shown in the active target network diagram (Figure 4B), the top four metabolites in the network based on degree value are Luteolin, Dehydrodiisoeugenol, Apigenin, and Naringenin. To identify genes related to neuroinflammation-associated depression, we conducted a PPI network analysis using the STRING database. The resulting PPI network had 13 nodes and 25 edges (Figure 4C). To visualize the relationships within the PPI network, the data were fed into Cytoscape 3.7.1, and degree values were computed using the CytoHubba tool. The analysis revealed that CXCL8, ICAM1, NOS2, SELP, TNF, IL6, APP, ACHE, MAOA, ADA were the 10 targets with the highest degree values. Hence, these targets were considered critical in the ECS-mediated treatment of neuroinflammation-associated depression (Figure 4D).

#### 3.2.3 GO analysis of putative targets

**FIGURE 5:**
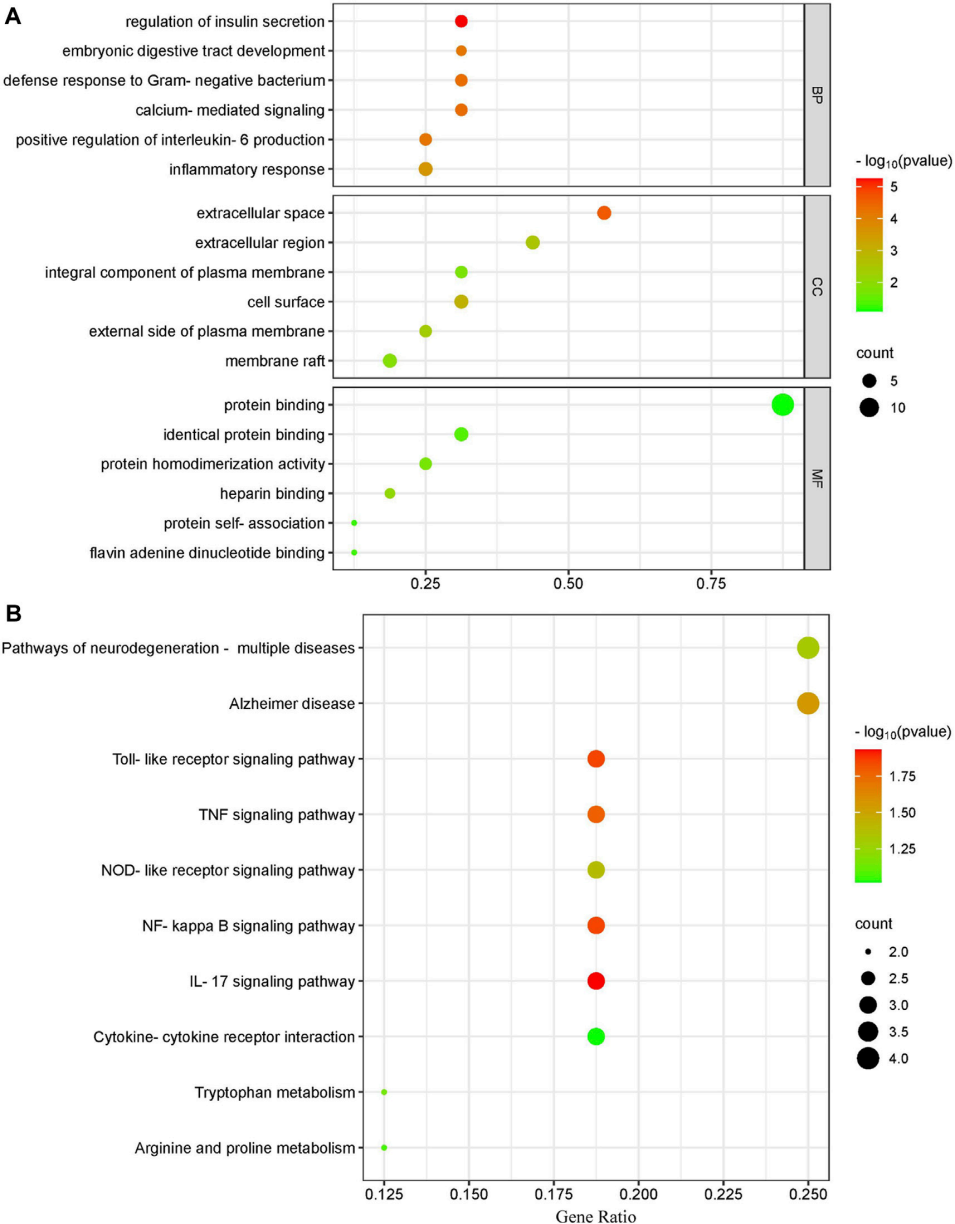
Gene ontology terms and KEGG pathway enrichment of core candidate targets of against neurionflammmation depressin **(A)** GO of core candidate of ECS against neurionflammation depreesion the terms in each Go category with *p* value < 0.05 were selected BP biological process CC, celluler components MF moleculer function **(B)** KEGG pathway analysis of major targets the top-17 pathway that had significant changes of *p* value < 0.05 were identified *X*-axis showes enrichment and *Y*-axis Shows involved pathways Count and *p* value are shown on right.

Using the Cluster Profiler program, we conducted KEGG and GO pathway enrichment for the major targets to determine the molecular systems affected by ECS in neuroinflammation-associated depression treatment. GO enrichment categories included BP, CC, and MF. For each dataset, we selected the top six terms (Figure 5A). For BP, regulation of insulin secretion (GO:0050796), calcium-mediated signaling (GO:0019722), defense response to Gram-negative bacterium (GO:0050829), positive regulation of interleukin-6 production (GO:0032755), embryonic digestive tract development (GO:0048566), inflammatory response (GO:0006954).

For CC, extracellular space (GO:0005615), cell surface (GO:0009986), extracellular region (GO:0005576), external side of plasma membrane (GO:0009897), membrane raft (GO:0045121), integral component of plasma membrane (GO:0005887).

The top MF terms included heparin binding (GO:0008201), protein homodimerization activity (GO:0042803), identical protein binding (GO:0042802), flavin adenine dinucleotide binding (GO:0050660), protein self-association (GO:0043621), protein binding (GO:0005515).

#### 3.2.4 Pathway analysis of ECS targets

In addition to the IL-17 signaling pathway (has04060) and TNF signaling pathwahashsa (04668), the nuclear factor kappa B (NF-κB) signaling pathway (hsa04064) and T-cell receptor signaling pathway (has04660) were selected from a total of 37 KEGG pathways derived from the top 10 most significantly enriched KEGG pathways. The correlation between the pathways and hub targets was then revealed by constructing a target gene–pathways network (Figure 5B). The top 10 significant pathways were categorized into three functional modules related to depression, T-cell activation, and cellular apoptosis. Detailed information on the pathway analysis results is provided in Supplementary Table S2. However, further research is necessary to clarify the exact mechanisms through which ECS exerts its antidepressant effect.

### 3.3 Effect of ECS on the body weight and behavior of CORT-induced depression mice

**FIGURE 6:**
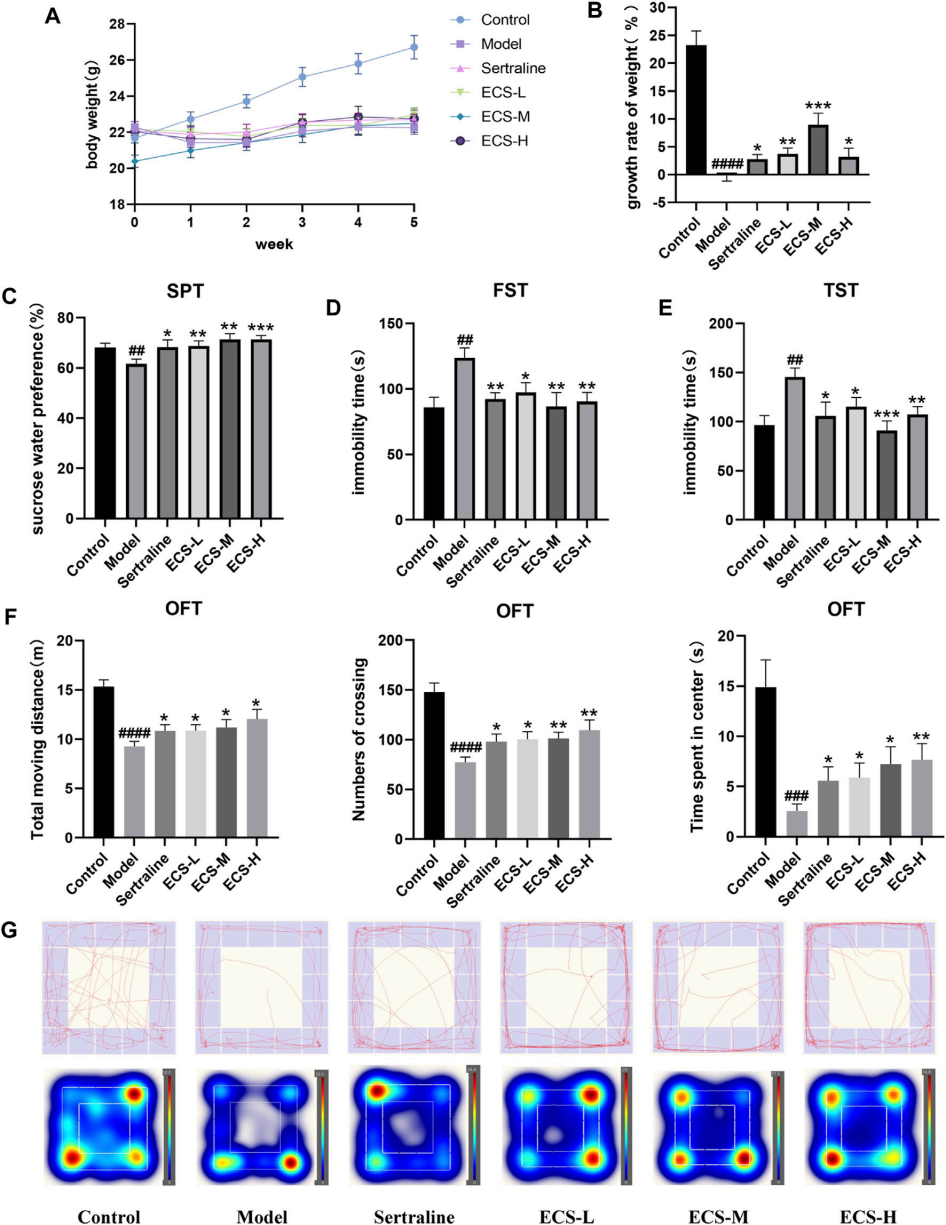
CORT induced depression like behavior in mice **(A,B)** Body weight change and growth rate **(C)** Sucrose preference test (SPT) **(D)** Forced swimming test (FST) **(E)** Tail suspension test (TST) **(F)** OFT data analysis **(G)** of track diagram and hetmap #*p* < 0.05,##<0.01, ###*p* < 0.001,####*p* < 0.0001 compared with Model group result are presented as Mean ± SEM, n = 9–10.

To validate the therapeutic effect of ECS on depression, we constructed a mouse model of CORT-induced depression. We monitored the body weights of mice weekly to assess the impact of sertraline and ECS. No significant differences in the body weight of the mice were observed before the experiment (Figure 6A). However, after 5 weeks, the body weight of the mice in the model group significantly decreased compared with that of the control mice (*p* < 0.0001), suggesting that CORT could potentially affect the body weight of growing mice.

Mice from all treatment groups grew faster than model mice during treatment (*p* < 0.05). However, the mice in the ECS-M group gained significantly more weight than those in the other groups (*p* < 0.05). The results demonstrate that ECS has a similar effect to that of sertraline in reversing the CORT-induced weight changes, significantly accelerating the growth rate of mice to normal levels (Figure 6B).

SPT, FST, TST, and OFT were used to assess depression-like behavior (Figures 6C–G). The percentage of sucrose water ingested by mice in the different groups at the start of SPT did not differ significantly (*p* > 0.05). The consumption of sucrose water by the model group was significantly lower (*p* < 0.01) than that of the control group. The immobility time in the model group significantly increased throughout FST and TST (*p* < 0.01), whereas crossing movements, time spent in the center, and overall movement distance in OFT significantly decreased (*p* < 0.01). Thus, the successfully established model demonstrated that mice with CORT-induced depression had a lower preference for sucrose and exhibited less movement than the control mice. In contrast to the model group mice, those from treatment groups showed significantly increased sugar water consumption during SPT as well as greater movement distance, crossing times, and accumulated time in the center during OFT (*p* < 0.05). Furthermore, their immobility times in TST and FST were significantly reduced (*p* < 0.01). These results demonstrate the amelioration of CORT-induced depression via ECS treatment. Notably, mice in the ECS-H treatment group exhibited higher autonomic activity and a stronger desire to explore new environments during SPT and OFT than those treated with sertraline.

Collectively, these results indicate that ECS has an efficacy similar to that of sertraline in ameliorating depression-related behavior in mice with CORT-induced depression.

### 3.4 Effect of ECS on neuroinflammation in the hippocampi of mice with CORT-induced depression

According to the drug–target–disease network, NOS2, a microglial polarization marker involved in regulating inflammatory factors, is a crucial target for the antidepressant effects of ECS. After being activated in the brain, microglia can undergo polarization and participate in both neurodegeneration and healing processes. These observations prompted us to explore whether ECS can regulate microglial phenotype by affecting microglial activation.

**FIGURE 7:**
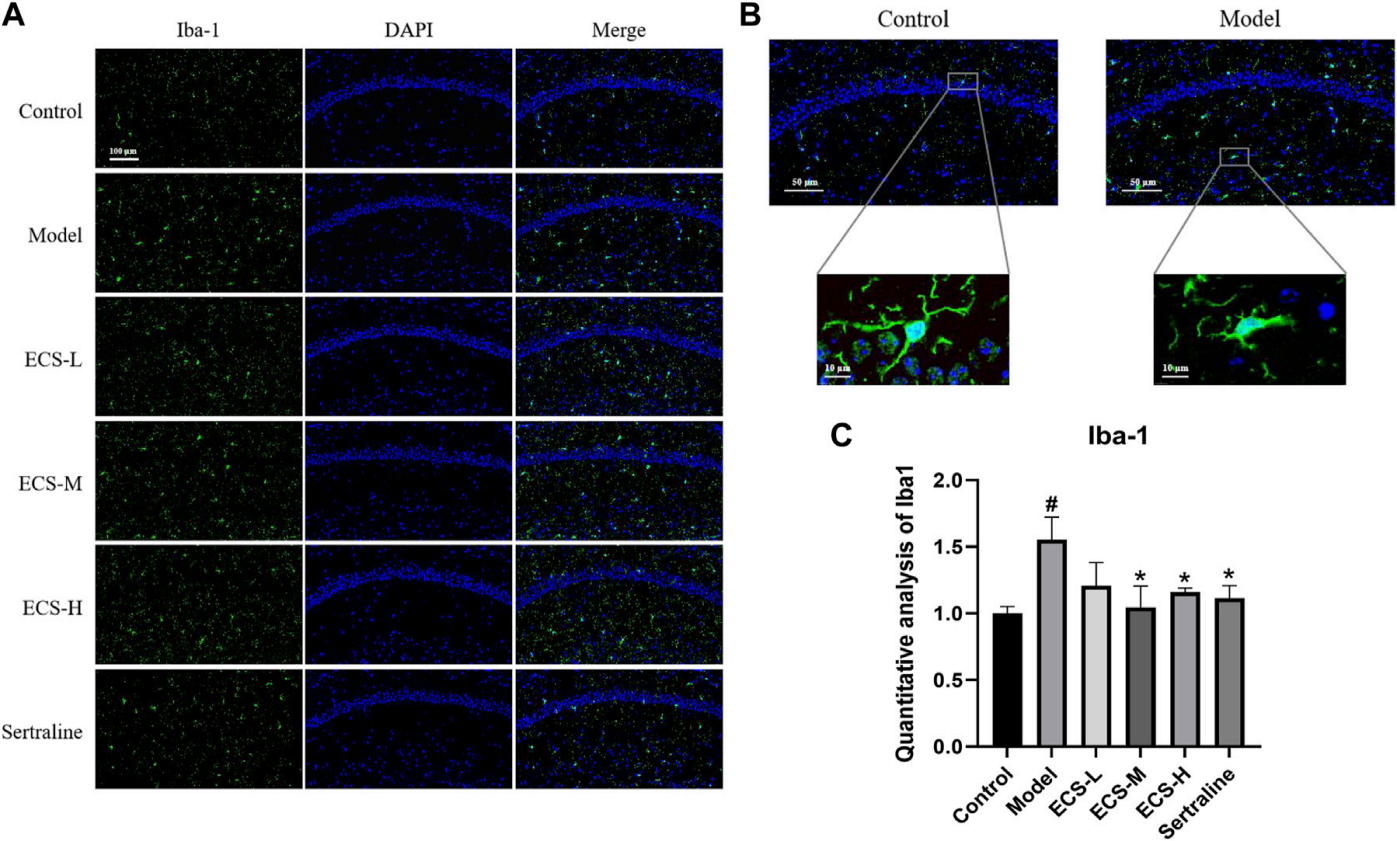
ECS can alleviated the activation of microglia in hipppocampal tissues of CORT-treated mice **(A)** Representative photos of lba-1 positive cells (green) in the hippocampus of mice Scale bar = 100um **(B)** Morpholgy of lba-1 cells in the mouse hippocampus **(C)** Quantifiction of lba-1 immunostaining hippocampus of differnt groups mice #*p* < 0.05 compared with control group; *p < 0.05 compared with model group Result are presented as Mean ± SEM, n = 3.

To clarify the effect of ECS on microglial activation in mice with CORT-induced depression, we used immunofluorescence staining to detect the microglia-specific marker Iba-1 and assess the quantity of Iba-1-positive cells within hippocampal microglia and their activation status. Figure 7B shows that microglia in the control group exhibited a branched morphology with a small cell body and branches extending in all four directions. In the model group, microglia transformed into an amoeba-like morphology, characterized by a swollen cell body and shortened processes. The hippocampi of control group mice showed only a few activated Iba-1-positive cells (Figures 7A, C). In contrast to that of the control mice, the quantity of Iba-1-positive cells in the hippocampi of model group mice significantly increased (*p* < 0.05), whereas that of sertraline-treated mice, as well as that of the ECS-M and -H groups, significantly decreased (*p* < 0.05).

**FIGURE 8:**
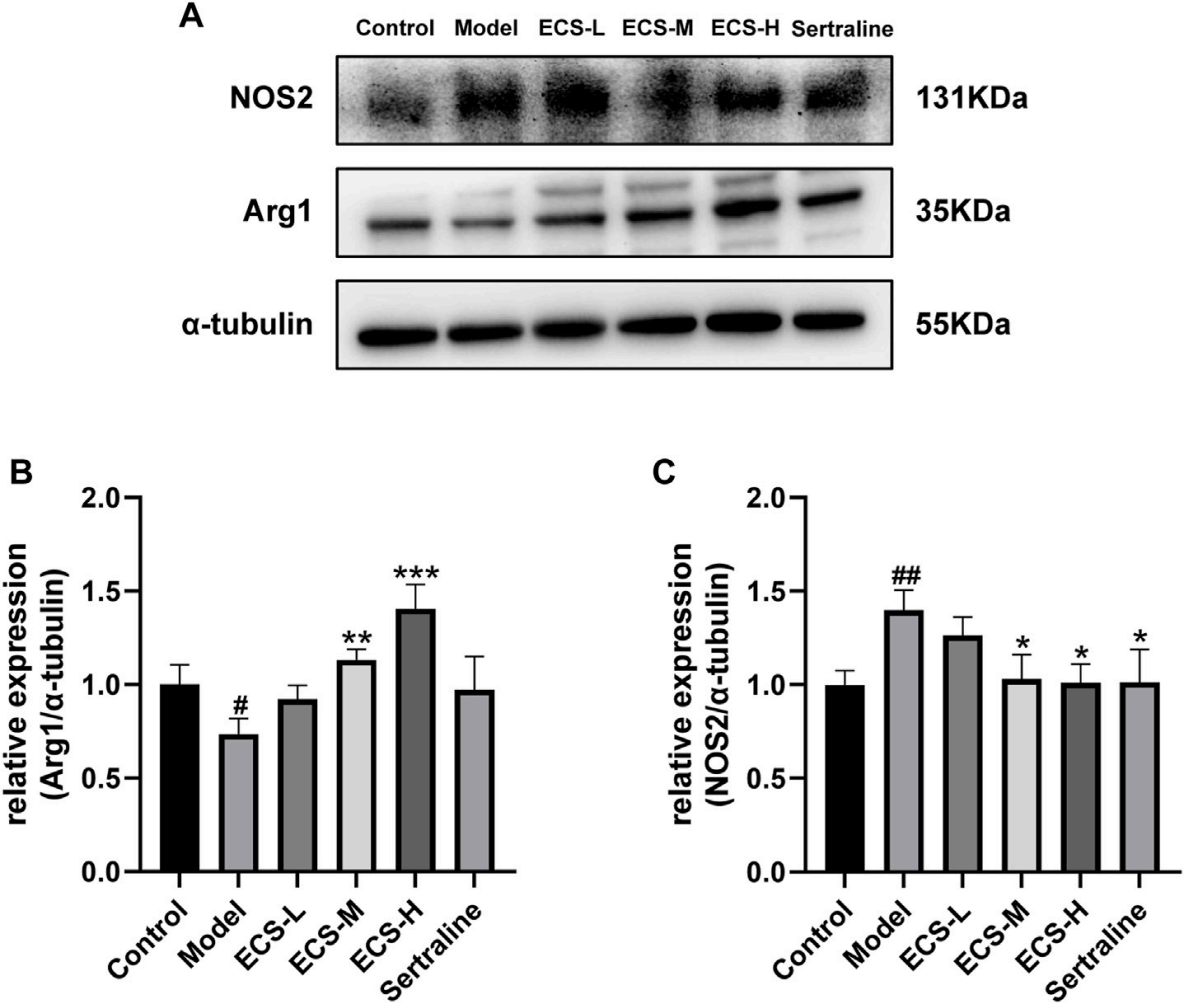
ECS can regulation of the microglial M1/M2 polarized phenotype attenuates neuriomflammation in hippocampus tissues of CORT-treatment mice **(A)** the protein leavels of NOS2 and Arg1 were detected by western blot and alph tubulin was used as the internal reference protein.statistical analysis of NOS2 and Arg2 proteins in **(B,C)** #*p* < 0.05, ##*p* < 0.01 commpared with control group; *p < 0.05,**p < 0.01,***p < 0.001 copared with model group Results are presentd as Mean ± SEM, n = 6-7

**FIGURE 9:**
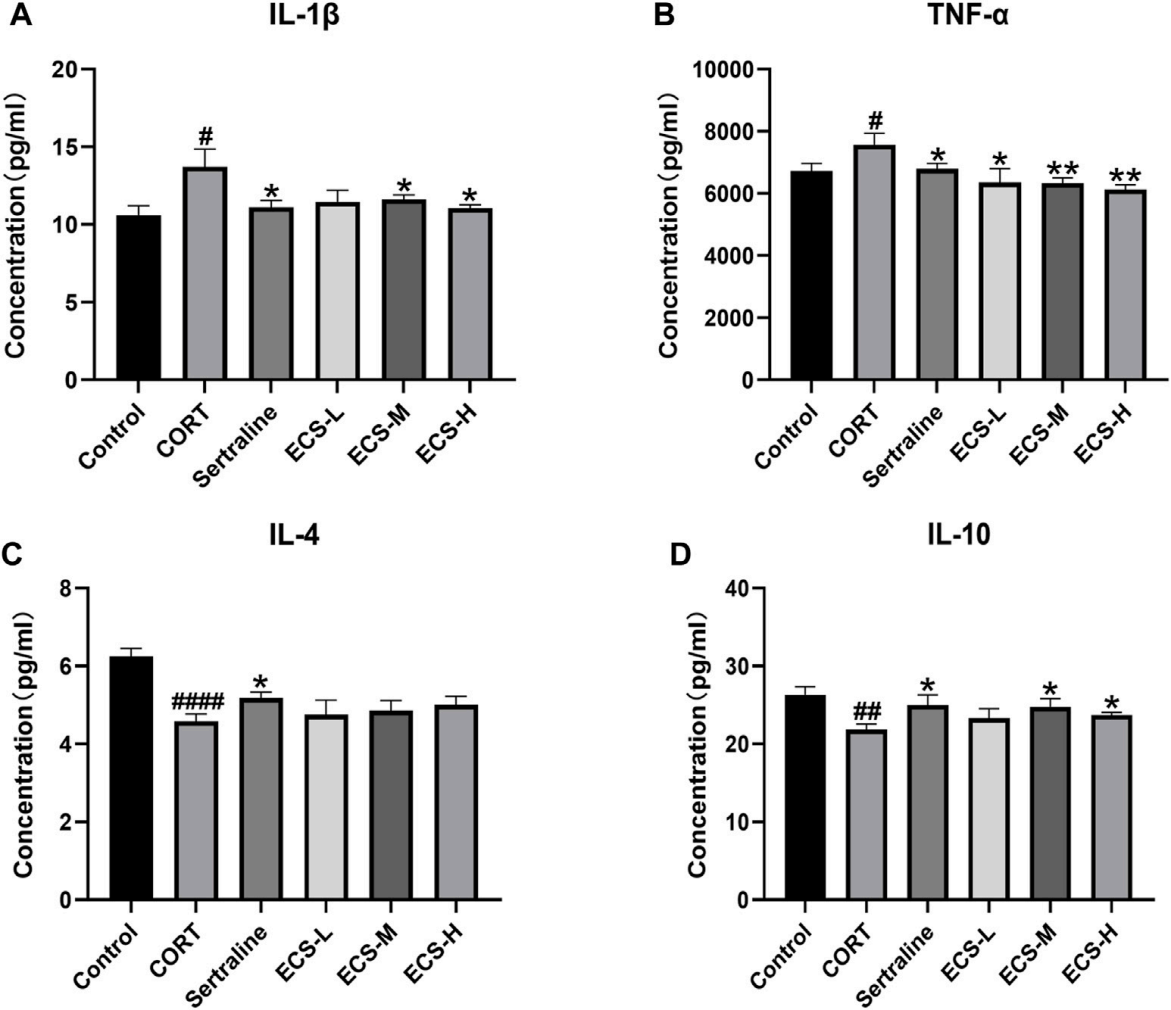
ECS can improved hippocampus inflammation in hippocampus tissues of CORT-treated mice.CORT treatment resulted in higher leavels of pro-inflammatory cytokines, and ECS decreased the expression of IL-1β **(A)** and TNF-α **(B)** compared to the Model group **(D)** ECS Significantly increased the expression of anti-inflammtory cytokined IL-10 in the hippocampus of CORT induced mice but the expression of IL-4 did not changes sihgnficantly **(C)**.#*p* < 0.05.##*p* < 0.01,####*p* < 0.0001 compared with control group; *p < 0.05,**p < 0.01 compaerd with model group Results are presented as Mean ± SEM, n = 7.

These findings demonstrate that ECS is capable of effectively inhibiting microglial activation. To further assess the effect of ECS on microglial polarization, western blotting was conducted to investigate the expression of microglial M1 and M2 polarization markers NOS2 and Arg1 in the mouse hippocampus (Figure 8). Compared to those of the control mice, Arg1 levels in the hippocampi of model group mice were significantly lower (*p* < 0.05), whereas NOS2 levels were significantly higher (*p* < 0.01). Our findings suggest that mice treated with CORT may exhibit depression-like behaviors, which could be attributed to increased microglial polarization. Additionally, the hippocampal levels of Arg1 in the mice of the ECS-M and -H treatment groups were significantly higher than those of model group mice (*p* < 0.05); however, NOS2 expression levels in the hippocampi of mice in the ECS-H and -M treatment groups were significantly lower (*p* < 0.05) than those in model group mice. These results suggest that ECS treatment reduced microglial M1 polarization in the hippocampi of mice with CORT-induced depression while inducing M2 polarization. As microglial activation primarily induces nerve injury via enhanced inflammation, hippocampal samples from each group were subjected to ELISA to determine IL-4, IL-10, IL-1β, and TNF-α levels (Figure 9). The anti-inflammatory cytokines IL-10 and IL-4 were significantly lower (*p* < 0.01) in the hippocampi of model group mice than in those of control mice. However, the levels of pro-inflammatory cytokines IL-1β and TNF-α were significantly high (*p* < 0.05). These findings imply that inflammatory factor production is linked to the development of depression in these mice. TNF-α and IL-1β levels were significantly lower (*p* < 0.01 and *p* < 0.05, respectively) in the ECS-M and -H groups than in the model group, whereas IL-10 was significantly higher (*p* < 0.05). ECS-L group mice exhibited reduced TNF-α expression levels, along with other inflammatory factors. However, the changes in IL-4 were not statistically significant. Overall, our findings indicate that a sufficient dose of ECS can decrease inflammation levels in the hippocampi of CORT-treated mice, with ECS-H treatment outcomes being similar to those of sertraline treatment.

### 3.5 Effect of ECS on NF-κB signaling in the hippocampi of mice with CORT-induced depression

**FIGURE 10:**
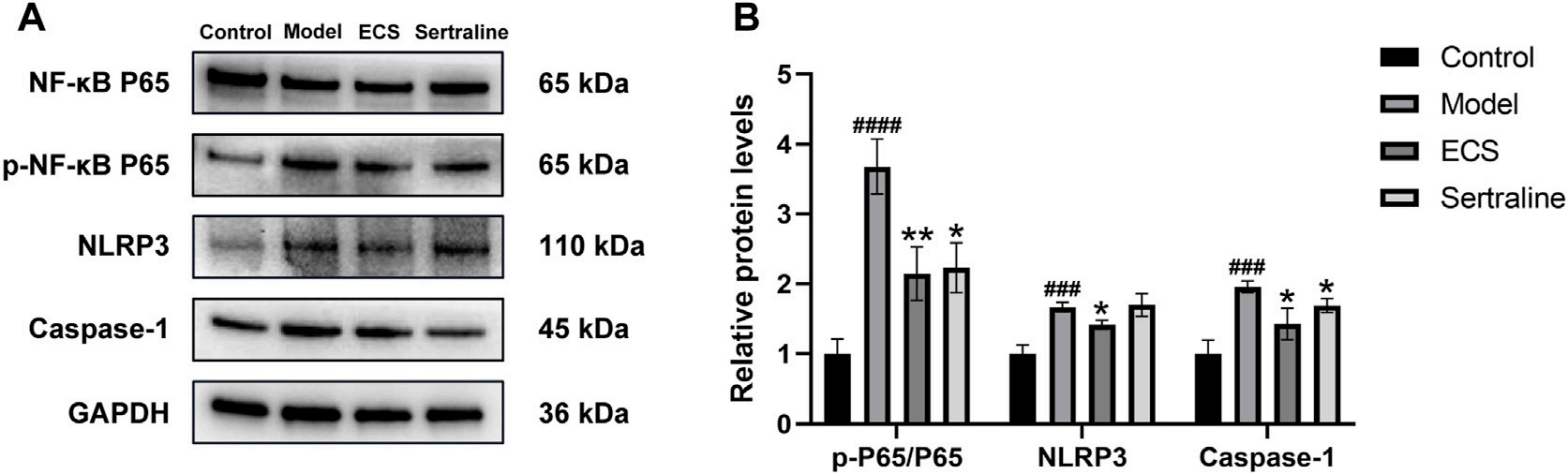
ECS can affect the expression of proteins involved in the NF-kB-NLRP3 signaling pathway in the hippocampus **(A)** the protein leavel of NLRP3,Caspase-1,p-NF-kB P65 and NF-kB p65 were detected by western blot **(B)** the p-p65/p65 represent the ratio of NF-kB P65 Phosphorylated versus non-phosphorylated proteins, GAPDH was used as the internal refernce protein statistical analysis of NLRP3,Caspase-1,p-p65/p65 proteins.###*p* < 0.001,####*p* < 0.0001 compared with control group *p < 0.05,**p < 0.01 compared with model group.results are represent as Mean ± SEM,n = 6.

In neuroinflammation-associated depression, NF-κB pathway is one of the key pathways targeted by ECS, and it is implicated in various central nervous system diseases (Zhang et al., 2013). It plays an essential role in regulating the production of inflammatory mediators and in microglial polarization. Therefore, it is of great importance to determine whether the NF-κB signaling pathway is affected by ECS in exerting its antidepressant-like effects. The western blotting results (Figure 10) showed that, compared with the levels in the control mice, NLRP3 (*p* < 0.001), caspase-1 (*p* < 0.001), and p-NF-κB P65/NF-κB P65 (*p* < 0.0001) levels were significantly upregulated in the model group mice. After the 5-week intervention, compared to those in the model group, NLRP3 (*p* < 0.05), caspase-1 (*p* < 0.05), and p-NF-κB P65/NF-κB P65 (*p* < 0.01) levels were significantly downregulated (*p* < 0.05) in the ECS-H group mice. Overall, the western blotting results confirmed the findings obtained by network analysis, indicating that ECS can regulate the NF-κB-NLRP3 inflammation pathway in treating depression.

## 4 Discussion

Depression is a mental disorder characterized by changes in the central nervous system, and its incidence has increased across all age groups in recent years, imposing a significant burden on healthcare systems worldwide (Whiteford et al., 2013). Approximately one-third of depressed patients do not respond to traditional antidepressants, and the effectiveness of low-dose antidepressants is only 20%–40%, with an increased risk of side effects at higher doses. It has prompted extensive research into developing effective alternative drug treatments for depression (Collins et al., 2011).

In recent decades, researchers have discovered that various herbal medicines have potent antidepressant-like effects with few adverse reactions (Yao et al., 2020). Thus, Chinese herbal medicines and their active ingredients have become the focus of depression drug development research. Previous studies confirmed that ECS exerts neuroprotective effects via activating the p38 MAPK/CREB pathway, inhibiting neuronal apoptosis, and repairing damaged neurons (Tian et al., 2014). The p38 MAPK signaling pathway is closely associated with inflammation (Lim and Tesch, 2012), suggesting that ECS can treat neurological diseases by alleviating neuroinflammation. Neuroinflammation is a serious immunomodulatory complex disease. In addition to their role in immune function, microglia are also closely linked to depression occurrence. Treating brain inflammation with novel drugs derived from natural sources is a crucial depression treatment strategy.

Using LC-MS/MS analysis, we identified the principal metabolites of ECS, including several natural metabolites with antioxidant and anti-inflammatory functions, such as phloridzin, phlorizin, and ursolic acid. Phloridzin is a glucoside of phloretin, a dihydrochalcone belonging to the flavonoids. It possesses various pharmacological properties, including anti-diabetic (Kobori et al., 2012; Mei et al., 2016), antioxidant (Liu et al., 2021), and anti-inflammatory (Tian et al., 2017) properties. ECS recognizes these two metabolites: phloridzin and phloretin. According to research, phloretin exerts pharmacological effects by acting on the NF-κB signaling pathway and downregulating inflammatory mediators (Habtemariam, 2023). In contrast, phlorizin ameliorates cognitive deficits by alleviating oxidative stress and neuroinflammation in an Alzheimer’s disease rat model (Tian et al., 2018). Ursolic acid is a pentacyclic tricarboxylic acid compound with neuroprotective effects; it can inhibit inflammatory factors’ overexpression and has a therapeutic effect on neurodegenerative diseases (Ramos-Hryb et al., 2017; Chen et al., 2020).

Network and component target function analyses revealed that luteolin, dehydrodiisoeugenol, apigenin, and naringenin rank high among ECS metabolites. Naringenin and apigenin have been discovered in *Cynomorium songaricum* Rupr. (Cui et al., 2013), and they are believed to possess neuroprotective effects. It has been reported that naringenin can penetrate the blood-brain barrier *in vivo* (Youdim et al., 2004), indicating its potential as a therapeutic agent for neurodegenerative diseases. Apigenin, a natural flavonoid, effectively reverses the depression-like behavior of LPS mice by regulating NF-κB activation in the prefrontal cortex and has anti-inflammatory properties (Li et al., 2015). Luteolin is a flavonoid compound that protects mouse neurons and alleviates depression-like behavior in CUMS mice via the Sirt1/NF-κB/NLRP3 signaling pathway (Xie et al., 2023). Dehydrodiisoeugenol, which play anti-inflammatory and antioxidant roles via regulation of canonical pathways such as nuclear factor kappa B (NF-κB) in microglia and microphages (Qiburi et al., 2021). Regeneration of nerves is a complex process involving multiple pathways. Additionally, some metabolites identified by LC-MS/MS analysis are polyphenols with a widespread distribution. Therefore, due to the limitations of the network analysis used in this study, further experimental verification is required to determine the pharmacological activity of its specific metabolites.

Network analysis revealed the relationships between drugs and targets at the biological regulatory network level. We focused mainly on the regulatory effect of ECS on microglia during depression treatment. From the analysis, 14 potential treatment targets for ECS-mediated anti-neuroinflammation depression were identified. According to studies, the hippocampus plays a crucial role in depression. It is a stress-sensitive structure vital for cognitive and spatial memory (Austin et al., 2001; Price and Drevets, 2010). Moreover, it regulates the hypothalamic-pituitary-adrenal (HPA) axis stress response. Glucocorticoid receptors are highly expressed in the hippocampus; prolonged exposure to high glucocorticoid levels can impair the functions of these receptors, leading to HPA axis dysregulation and hyperactivity and hippocampal neuronal damage. Accordingly, the hippocampus is closely related to the pathology of depression and the depression model discussed in this paper. To explore the anti-neuroinflammation role of ECS in depression, we selected hippocampus tissue and confirmed that ECS could reduce neuroinflammation in the hippocampus and protect the central nervous system. ECS exerts its antidepressant-like effects specifically by regulating the polarization and activation of microglia. Important pathophysiological mechanisms of depression include hippocampal inflammation and excessive microglial activation (Ricklin and Lambaris, 2013). NOS2, a key target identified from network analysis, is a marker of M1 microglial polarization. Microglia can polarize into an M1 or M2 phenotype, associated with different gene expression profiles and functions. During inflammation, microglia polarize to the M1 state to produce pro-inflammatory cytokines, a process called classical activation (Butovsky et al., 2006). Alternative activation is a process in which M2 microglia express Arg1, suppress inflammation, and release neurotrophic factors to promote neurological recovery (Szczepanik et al., 2001). Depressed patients have dysregulated M1 and M2 polarization. Persistent stress can promote M1 polarization to produce the pro-inflammatory cytokines IL-1β, IL-6, and TNF-α and reactive oxygen species, triggering a local inflammatory response and impairing cognitive performance. Furthermore, releasing inflammatory factors may reduce the severity of the inflammatory response and promote nerve tissue repair by enhancing M2 polarization (Francos-Quijorna et al., 2016). Therefore, promoting M2 phenotype polarization is one strategy for treating the central nervous system (Campos et al., 2020).

To elucidate the action mechanism of ECS during depression therapy, enrichment analyses of functional GO and KEGG pathways were conducted. We focused on the anti-neuritic effect of ECS on microglia regulation. NF-κB is a crucial pro-inflammatory transcriptional regulator that drives the generation of diverse pro-inflammatory cytokines, including IL-1β, IL-6, and TNF-α (Mitchell and Carmody, 2018). NF-κB inhibitors reduce NLRP3 inflammasome activation in a dose-dependent manner, suggesting that NF-κB plays an essential role in the inflammatory response (Zhou et al., 2011). NF-κB functions not only as an activation signal for the NLRP3 inflammasome but also as a transcription factor that regulates NLRP3 expression, thereby jointly mediating the occurrence and development of depression. Overactivation of the NLRP3 inflammasome causes the overproduction of pro-inflammatory cytokines, such as IL-18 and IL-1β, via the cleavage of pro-caspase-1 by NLRP3 and ASC, thereby activating downstream signaling pathways and ultimately contributing to an inflammatory cascade that leads to depression. These observations indicate that the NF-κB-NLRP3 signaling pathway plays an essential role in inducing the inflammatory response and can be a crucial target in treating depression. Furthermore, the present study revealed that CORT-induced depression in mice resulted in notable depression symptoms, increased levels of pro-inflammatory factors, including IL-1β and TNF-α, and an increase in NF-κB phosphorylation. In contrast, ECS therapy reduced TNF-α, IL-1β, p-NF-κB/NF-κB P65, NLRP3, and caspase-1 levels, attenuating neuroinflammation and depressive behavior. Our results demonstrated the antidepressant potential of ECS, which is closely linked to the inhibition of NF-κB-NLRP3 pathway-mediated hippocampal inflammation. In the future, it is intended to conduct *in vitro* experiments further to validate the antidepressant mechanism of action of ECS. Additionally, blood metabolites analysis will be performed to gain a better understanding of the active metabolites associated with ECS. These future research endeavors will contribute to a more comprehensive understanding of ECS and provide empirical evidence of its antidepressant effects, laying the scientific foundation for subsequent clinical research and applications.

## 5 Conclusion

According to our findings, ECS exerts its antidepressant effects by inhibiting neuroinflammation. Specifically, it is found that ECS suppresses the NF-κB-NLRP3 pathway to regulate microglia’s M1/M2 polarization and reduce inflammation, thereby exerting antidepressant-like effects. Overall, our research provides significant insights into ECS’s effects and mechanisms in treating neuroinflammation-associated depression.

## Data Availability

The original contributions presented in the study are included in the article/Supplementary Material, further inquiries can be directed to the corresponding author.

## Footnotes

1
http://www.chemspider.com/Default.aspx

2
https://massbank.eu/MassBank/

3
https://old.tcmsp-e.com/tcmsp.php

4
http://swisstargetprediction.ch/

5
http://sea.bkslab.org/

6
http://lilab-ecust.cn/pharmmapper/

7 Pubmed2ensembl.

8
https://www.uniprot.org/

9
http://www.bioinformatics.com.cn/

10
https://String-db.org/

11
https://david.ncifcrf.gov/
