# Supplementary material for: Investigation of anti-depression effects and potential mechanisms of the ethyl acetate extract of Cynomorium songaricum Rupr. through the integration of in vivo experiments, LC-MS/MS chemical analysis, and a systems biology approach
Source: Front Pharmacol. 2023 Oct 25;14:1239197. doi: 10.3389/fphar.2023.1239197 (PMC10634308; doi:10.3389/fphar.2023.1239197)
Supplement: Supplementary file 3 [file Table1.DOCX]

**LC-MS analysis**

LC-ESI-MS/MS analysis was performed with a Thermo fisher U3000 ultra-high performance liquid chromatograph (UHPLC) equipped with an online degassing machine, quaternary gradient pump, column temperature chamber and automatic sampler and Q Exactive PlusTM Orbitrap MS system (Thermo Scientific, Waltham, MA, USA) equipped with a heated electrospray ionization (HESI) source. The chromatographic separation was carried out using Waters ACQUITY UPLC HSS T3 C18 column (2.1 mm × 100 mm, 1.8 μm; Waters Cor-poration, Milford, MA, USA). The UV detection of UHPLC fractions were performed by U3000 3D field DAD detector with wavelength coverages from 200nm to 400nm.. The analytical column was maintained at 30 °C with an injection volume of 5μL. Gradient elution was carried out with water with 0.1% (v/v) formic acid in water (solvent B) and acetonitrile (solvent A). The flow rate was 0.2 ml/min, and gradient elution was as follow: 0-10min, 100% B; 10-20min, 100%-70% B; 20-25min, 70%-60% B; 25-30min, 60%-50% B; 30-40min,50%-30% B; 40-45min, 30%-0% B; 45-60min, 0% B; 60-60.1min, 0%-100% B; 60.1-70min, 100% B. Positive and negativeion mode in the range of m/z 100-1500 was set for the MS ananlysis. Other working MS parameters were sheath gas flow of 40 arb, auxiliary gas flow rate of 15 arb, capillary temperature of 320 °C, Aux gas heater temperature of 350 °C, positive spray voltage of 3.2 kv. The resolution of MS is 70000, and the resolution of MS/MS is 17500. The identification of unknown compound was performed by Compound discover 3.2.0.305, with mzcloud and mzVault databases.
